# Protection of Mitochondrial Potential and Activity by Oxyprenylated Phenylpropanoids

**DOI:** 10.3390/antiox12020259

**Published:** 2023-01-23

**Authors:** Francesco Epifano, Salvatore Genovese, Lucia Palumbo, Chiara Collevecchio, Serena Fiorito

**Affiliations:** Department of Pharmacy, University “Gabriele d’Annunzio” of Chieti-Pescara, 66100 Chieti Scalo (CH), Italy

**Keywords:** auraptene, 7-isopentenyloxycoumarin, mitochondria, organophosphates, oxyprenylated phenylpropanoids, pesticides

## Abstract

A series of five naturally occurring oxyprenylated phenylpropanoids, namely, the coumarins auraptene (7-geranyloxycoumarin) **1** and 7-isopentenyloxycoumarin **2**, and the coumaric acid and ferulic acid derivatives, 4’-isopentenyloxycoumaric acid **3**, boropinic acid **4**, and 4’-geranyloxyferulic acid **5** were tested for their effects on mitochondrial functionality using the organophosphate pesticides glyphosate and chlorpyrifos, and resveratrol, as the reference. While not showing an appreciable in vitro antioxidant activity, and virtually no or a little effect on the viability of non-cancer cell lines BEAS-2B and SHSY-5Y, all phytochemicals exhibited a marked protective effect on mitochondrial potential and activity, with values that were comparable to resveratrol. Auraptene **1** and 7-isopentenyloxycoumarin **2** were seen to be the most effective secondary metabolite to this concern, in particular in being able to completely abolish the decrease of mitochondrial potential induced by increasing concentration of both glyphosate and chlorpyrifos. All the compounds tested also exhibited a protective effect on mitochondrial activity. The potency displayed will shed more light on the molecular basis of the beneficial effects of auraptene, 7-isopentenyloxycoumarin, and structurally related oxyprenylated phenylpropanoids reported to date in the literature.

## 1. Introduction

**Figure 1:**
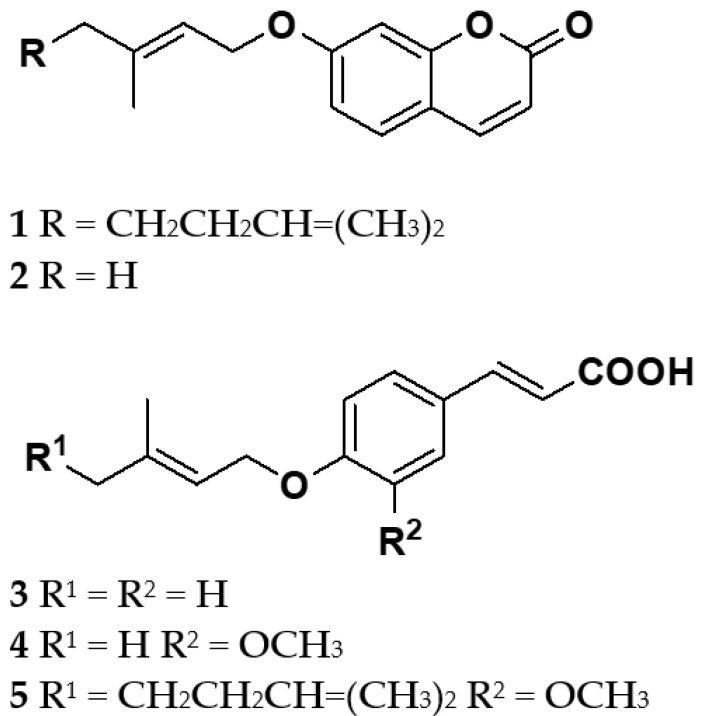
Structures of the phytochemicals under investigation. Compounds belong to two phenylpropanoids subclasses, such as *O*-prenylcoumarins (**1–2**), and cinnamic acid derivatives (**3–5**).

Oxyprenylated secondary metabolites, such as isopentenyloxy- (3,3-dimethylallyl) (C_5_), geranyloxy- (C_10_), and farnesyloxy- (C_15_) compounds, represent a family of rare natural products that were considered for years to be merely biosynthetic intermediates of the more widespread *C*-prenylated derivatives. These secondary metabolites have been recognized in the last twenty-five years as promising and valuable biologically active phytochemicals in several therapeutic areas such as oncology, neuroprotection, inflammation, stroke, cardiovascular disorders, microbial infections, dermatology, diabetes, obesity, metabolic syndrome, and several others. Until now, approximately 350 compounds have been isolated and structurally characterized from plants, primarily belonging to the families Rutaceae and Apiaceae, which in turn comprise several edible vegetables and fruits, fungi, protozoa, and bacteria. As a continuation of our ongoing studies aimed to gain further insights into the pharmacological profiles and potentialities of naturally occurring oxyprenylated secondary metabolites and their semisynthetic derivatives, we decided to investigate the effects of five selected sample plant compounds, namely auraptene **1**, 7-isopentenyloxycoumarin **2**, 4’-isopentenyloxycoumaric acid **3**, boropinic acid **4**, and 4’-geranyloxyferulic acid **5** on mitochondrial functionality stressed by the application of increasing concentrations (10–100 μM) of organophosphate pesticides such as glyphosate and chlorpyrifos. The chemical structures of the natural compounds under investigation in the present study are illustrated in Figure 1.

Auraptene **1** is widespread in plants belonging to the Rutaceae family comprising commonly consumed food (e.g., Citrus fruits) [1], 7-isopentenyloxycoumarin **2** has been, like **1**, mainly found in species belonging to the families of Rutaceae and Apiaceae [2], 4’-isopentenyloxycoumaric acid **3** has been isolated from the Brazilian tree *Esenbenckia hieronymi* Engl. (Rutaceae) [3], boropinic acid **4** has been originally extracted from the Australian shrub *Boronia pinnata* Sm. (Rutaceae) but also found in several edible rutaceous and apiaceous plant species [4], and finally 4’-geranyloxyferulic acid **5** has been originally obtained from the root bark extracts of the Australian small tree *Acronychia baueri* Schott (Rutaceae) and very recently its presence has been disclosed in commonly consumed food such as citrus fruits, quinoa, goji, spinach, and beet products [5,6,7].

Past literature reports about the biological activities displayed by the above-listed compounds refer mainly to the chemoprevention of several types of cancers (in particular those affecting the gastro-intestinal apparatus, such as the esophagus, stomach, and colon) by dietary feeding [8,9,10,11,12] as well as its neuroprotective [13,14,15];anti-inflammatory [16,17,18,19,20,21,22,23], anti-bacterial, and antifungal effects [24,25,26,27,28], lipid and sugar metabolism [3,29,30,31,32], and melanogenesis modulatory properties [33,34]. A survey of the biological properties of naturally occurring oxyprenylated phenylpropanoids was recently reported [35]. This indicates that, even if several subcellular structures (e.g., enzymes and membrane and nuclear receptor) have been investigated, very few data about the interaction of naturally occurring oxyprenylated phenylpropanoids and mitochondria have been described in the recent literature. The choice of this cell organelle was influenced by the fact that mitochondria are nowadays well recognized to play a pivotal role in the physiology and pathology of a plethora of severe acute and chronic syndromes affecting humans. Until now, mitochondria-targeted therapies have mainly focused on disorders evoked by mutations in mitochondrial and/or nuclear DNA for gene-encoding mitochondrial proteins, recent findings also indicate that mitochondrial dysfunction and alteration may be determinants of common pathologies, such as cancer, neurodegeneration, metabolic syndrome, heart failure, ischaemia–reperfusion injury, stroke, inflammation, and protozoal infections [36]. In this context, the mitochondria represent an important drug target for these highly prevalent diseases and have also become the subject of intense research efforts for site-specific drug delivery [37]. Although several therapeutic strategies aimed at normalizing mitochondrial functions have been being proposed since the very beginning of the new century, and a few agents have entered phase I, II, and III clinical trials, the research on this challenging topic is a field of current and growing interest. Finally, the interaction between these organelles and oxyprenylated phenylpropanoids, with the notable exception of some preliminary data for auraptene 1, are not reported in the literature. To this aim, in this manuscript, we reported on the antioxidant properties of these molecules and their capacity to modulate mitochondrial functions stressed by the application of known poisons such as glyphosate and chlorpyrifos.

## 2. Materials and Methods

### 2.1. Chemistry

Auraptene 1, 7-isopentenyloxycoumarin 2, 4′-isopentenyloxycoumaric acid 3, boropinic acid 4, 4′-geranyloxyferulic acid 5 were chemically synthesized following the already reported procedures [38]. Purity degree (≥97.6%) for all compounds was assayed by HPLC. All compounds were used after NMR characterization. Analytical data for compounds 1–5 were in full agreement with those already reported for the same samples [38]. All reagents, substrates, and solvents, including those employed for cell viability assays, have been purchased from Merck Sigma Aldrich (Darmstadt, Germany).

### 2.2. Analysis of Antioxidant Capacity by the ORAC Assay

The ORAC assay (oxygen radical absorbance capacity) was performed by a commercial kit (Cell Biolabs Inc., San Diego, CA, USA) according to the manufacturer’s instructions. Briefly, increased concentrations of the samples (25 µL, ranging 0–50 µM) was mixed with 150 μL of 40 nM fluorescein solution, and incubated at 37 °C for 30 min., followed by the addition of 25 μL of 153 nM 2,2’-azobis(2-amidinopropane) dihydrochloride (AAPH). The fluorescence intensity was read in a plate reader (Infinite F200 PRO, Sunrise, Tecan, Männedorf, Swiss) with an excitation wavelength of 485/20 nm and an emission filter of 530/20 nm and recorded every minute after addition of AAPH, for 60 min. The ORAC assay quantifies the inhibition of fluorescence produced by peroxyl radicals generated at a constant rate by thermal decomposition of AAPH. Integration of the area under the fluorescence decay curve was performed using the software Gen5. The antioxidant capacity was expressed in mM Trolox Equivalent (TE) calculated from the Trolox standard curve.

### 2.3. Cell Viability

Bronchial epithelial cells (BEAS-2B) (ATCC CRL9609) were purchased from Fisher Scientific (part of Thermo Fisher Scientific Waltham, MS, USA). SHSY-5Y (ATCC CRL2266) were obtained from Merck Sigma Aldrich (Darmstadt, Germany). Both cells were seeded at 3 × 10^4^ cells/well in a 96-well, allowed to attach overnight, and treated with increasing concentrations of compounds (0–50 µM) for 24 h. After the treatment, 10 μM 3-(4,5-dimethylthiazol-2-yl)-2,5-diphenyltetrazolium bromide (MTT; 5 mg/mL in phosphate buffered saline [PBS]) was added and incubated at 37 °C for 3 h. After removing the medium, 200 μL of isopropanol was added to dissolve the crystals. Absorbance was read at 550 nm in an ELISA plate reader (Sunrise, Tecan, Männedorf, Swiss), and the results expressed as relative changes with respect to the controls set as 100%. Each experimental step was carried out in six replicates.

### 2.4. Mitochondrial Destabilization

The effects of the compounds **1–5** and resveratrol used as the reference on mitochondria were evaluated as for their capacity to restore mitochondrial function after organophosphate pesticide (glyphosate and chlorpyrifos) treatment. Briefly, BEAS-2B cells (3 × 10^4^ cells/well in a 96-well) were treated with either glyphosate or chlorpyrifos and at two concentration levels (10 and 100 µM) in the presence or absence of the test compounds (50 µM). After 24 h of incubation, the changes in mitochondrial potential and mitochondrial reducing activity (MRA) were evaluated. The changes in the mitochondrial potential were detected by 5,5′,6,6′-tetrachloro-1,1′,3,3′tetraethylbenzimidazolylcarbocyanine iodide/chloride (JC-1), a cationic dye that exhibits potential-dependent accumulation in mitochondria, indicated by fluorescence emission shift from red (∼590 nm) to green (∼525 nm). MRA was assessed by the resazurin assay. To this aim, cells were incubated with resazurin (6 µM) in the presence and absence of the compounds (50 µM) and the fluorescence intensity evaluated over time (0–240 min), in a plate reader (Infinite F200 PRO, Sunrise, Tecan, Männedorf, Swiss), and the results were normalized to the total protein using the Bradford assay (Sigma) [39]. Each experimental step was carried out in six replicates.

### 2.5. Statistical Analysis

The same general procedure as already reported has been followed [40].

## 3. Results

Compounds **1–5** were chemically synthesized following the well validated route already reported in the literature [38]. Briefly, oxyprenylated coumarins auraptene **1** and 7-isopentenyloxycoumarin **2** have been obtained by etherification of the OH function of commercially available umbelliferone with geranyl or 3,3-dimethylallyl bromide in the presence of dry K_2_CO_3_ as the base in refluxing acetone for 2 h and purification by crystallization (*n*-hexane). Samples **3–5** were been synthesized in two steps from the commercially available parent p-coumaric and ferulic acids that were first converted into the corresponding methyl esters in refluxing methanol and in the presence of catalytic amounts of conc. H_2_SO_4_ for 12 h, then alkylated following the same route as described above, and finally hydrolyzed with NaOH 2 N in the same reaction vessel to provide after acid-base work-up and crystallization (*n*-hexane) of the desired adducts **3–5**. All compounds were obtained in yields > 96.7% in high purity, notably without the need of any chromatographic purification.

**Figure 2:**
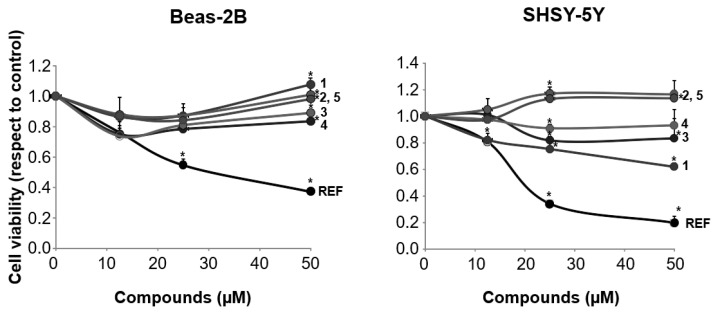
Effects of compounds **1–5** on cell viability in non-cancer cells (REF = resveratrol) BEAS-2B (human bronchial epithelium) and SHSY-5Y (neuronal cells). Values expressed as mean (*n* = 6) ± SD. * *p* < 0.01.

The preliminary assay we performed was to test the cytotoxicity of samples **1–5** by the MTT assay. To this aim we used two non-cancer cell lines, namely bronchial epithelial cells, BEAS-2B, and neuronal cells, SHSY-5Y. Compounds and reference resveratrol were assayed in the concentration range 10–50 μM. Results are reported in Figure 2.

**Figure 3:**
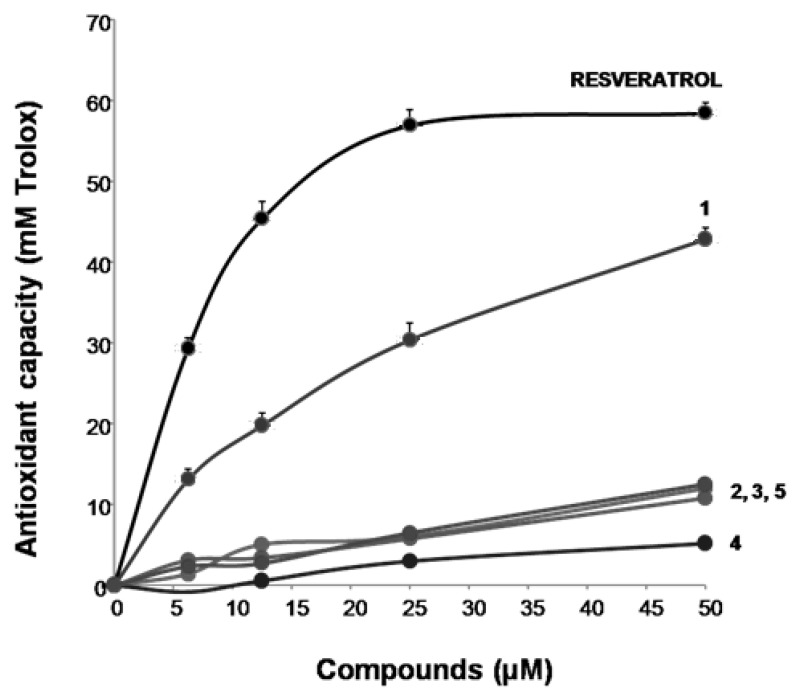
Antioxidant properties of compounds **1–5** (ORAC assay).

With these results in our hand, we could finally move to the next steps of our investigation, consisting in studying the effect of compounds **1–5** on mitochondrial destabilization and activity after treatment with the two pesticide chlorpyrifos and glyphosate. To this aim, BEAS-2B cells were used to accomplish these assays as it was seen as the only line practically unaffected by all chemicals under investigation in terms of cell viability. It is in fact clear from data reported in Figure 2 that neuronal cells SHSY-5Y were little affected by this concern, especially in the case of higher concentrations of auraptene **1** and 4′-isopentenyloxycinnamic acid **3.** Both glyphosate and chlorpyrifos are nowadays well known to deeply interfere with mitochondrial functions through extensive oxidative damage [41,42,43]. In particular, their main mitogen stimuli on mitochondria are represented by a massive induction of reactive oxygen species (ROS) production [44,45]. In detail, it has been seen how glyphosate, through the increased production of ROS (in particular hydrogen peroxide was found to be the main product of this oxidative burst) led to huge reductions in the proton gradient and ATP levels. On the other hand, chlorpyrifos was shown to induce via the mentioned ROS generation mitochondrial fragmentation via reduction of mitochondrial fusion protein mitofusin 1, a massive decrease of the membrane, and a marked decrease in ATP production. Thus, to exclude the possibility that compounds **1–5** could act as mere ROS and/or radical scavengers we decided to evaluate first the antioxidant capacities of the synthesized chemicals. This experiment was accomplished by the ORAC assay [46] expressing the total antioxidant capacity as Trolox equivalent (TE) calculated from the Trolox standard curve and using resveratrol as the reference. Results are reported in Figure 3.

Results reported in Figure 3 clearly suggest that only auraptene **1** is partially able to evoke a dose-dependent an antioxidant response (around 30% less than resveratrol at the highest concentration assayed, 50 μM). All the other compounds exhibited virtually no effect. These data are in line with what has been already highlighted in the literature showing how auraptene **1** had little antioxidant properties [47].

**Figure 4:**
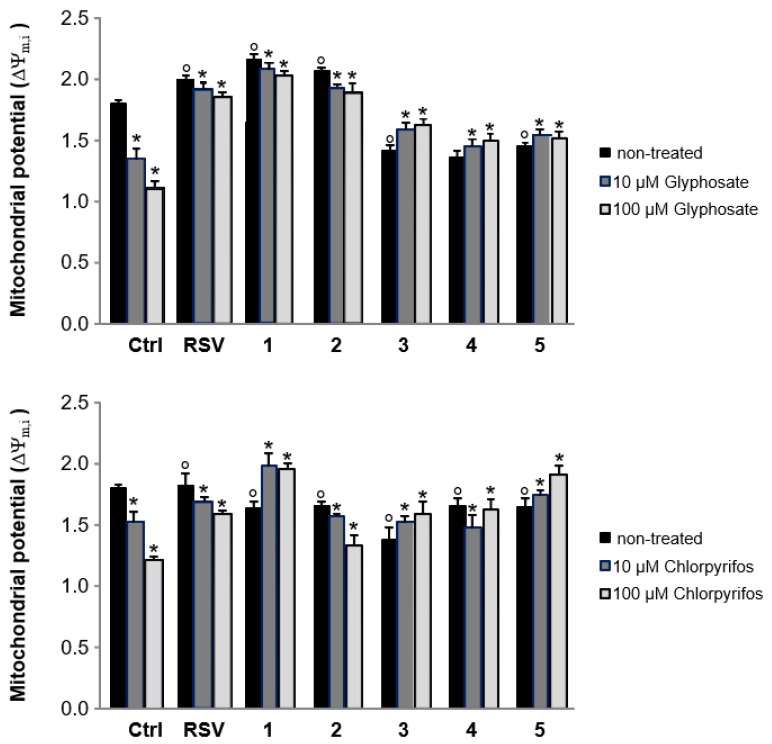
Effects of compounds **1–5** on mitochondrial destabilization (RSV = resveratrol), Values expressed as mean (*n* = 6) ± SD (* Significance non-treated vs. chlorpyrifos or glyphosate; ° Significance Ctrl vs. compounds).

**Figure 5:**
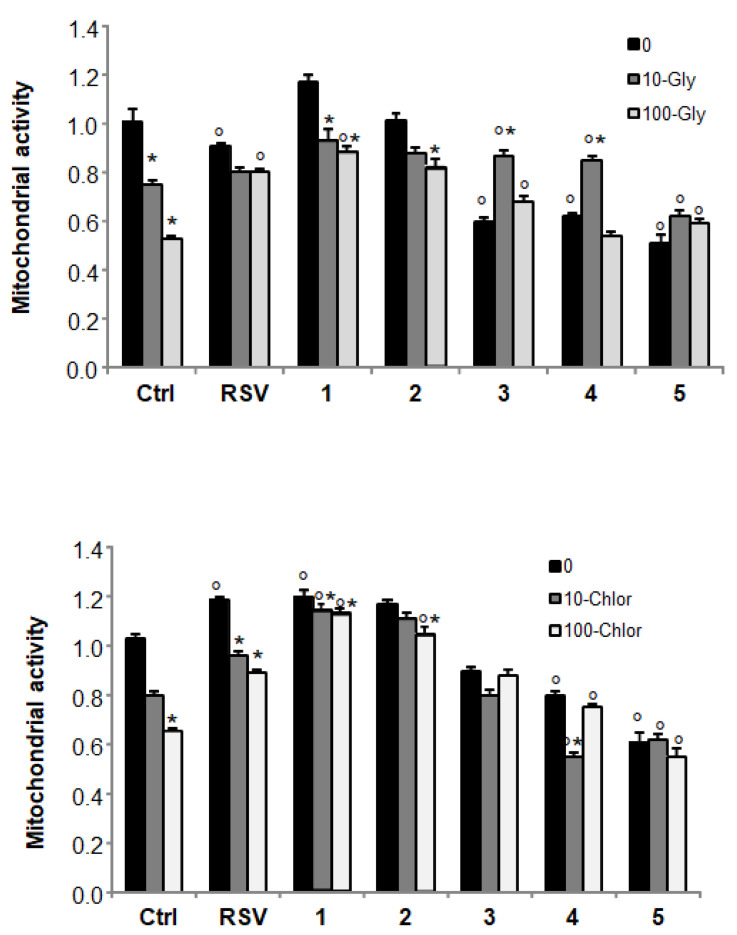
Effects of compounds **1–5** on mitochondrial activity (RSV = resveratrol) (* Significance non-treated vs. chlorpyrifos or glyphosate + compounds under investigation; ° Significance Ctrl vs. compounds).

We then moved to test the two organophosphate pesticides as mitochondrial poisons. Chlorpyrifos and glyphosate were tested at two concentration levels, namely 10 μM and 100 μM in the presence or absence of compounds **1–5** (50 µM). After 24 h of incubation, the changes in mitochondrial potential and MRA were evaluated. Results are reported in Figure 4 and Figure 5.

Data reported in Figure 4 and Figure 5 clearly indicate that treatment with both glyphosate and chlorpyrifos resulted in a substantial decrease of the mitochondrial potential, especially at the highest concentration value. Treatment with the reference compound resveratrol contributed to partially protect from the effect induced by these pesticides. These findings are absolutely in line with what have been reported about the protective effects of this stilbene in the same context [48]. For compounds under investigation, in most of cases a better performance than the reference resveratrol was recorded when the two organophosphates were administered to cells at their both concentrations in mixture with the compounds under investigation. Concerning mitochondrial activity, when sample oxyprenylated phytochemicals were administered to cells alone, only the oxyprenylated coumarins auraptene **1** and 7-isopentenyloxycoumarin **2** led to equal or increased activities with respect to the untreated controls.

When these same compounds **1** and **2** were applied in combination with chlorpyrifos and glyphosate in the range of the above indicated concentration levels, they were effective in restoring this parameter providing a performance comparable to that exhibited by the reference compound resveratrol.

**Figure 6:**
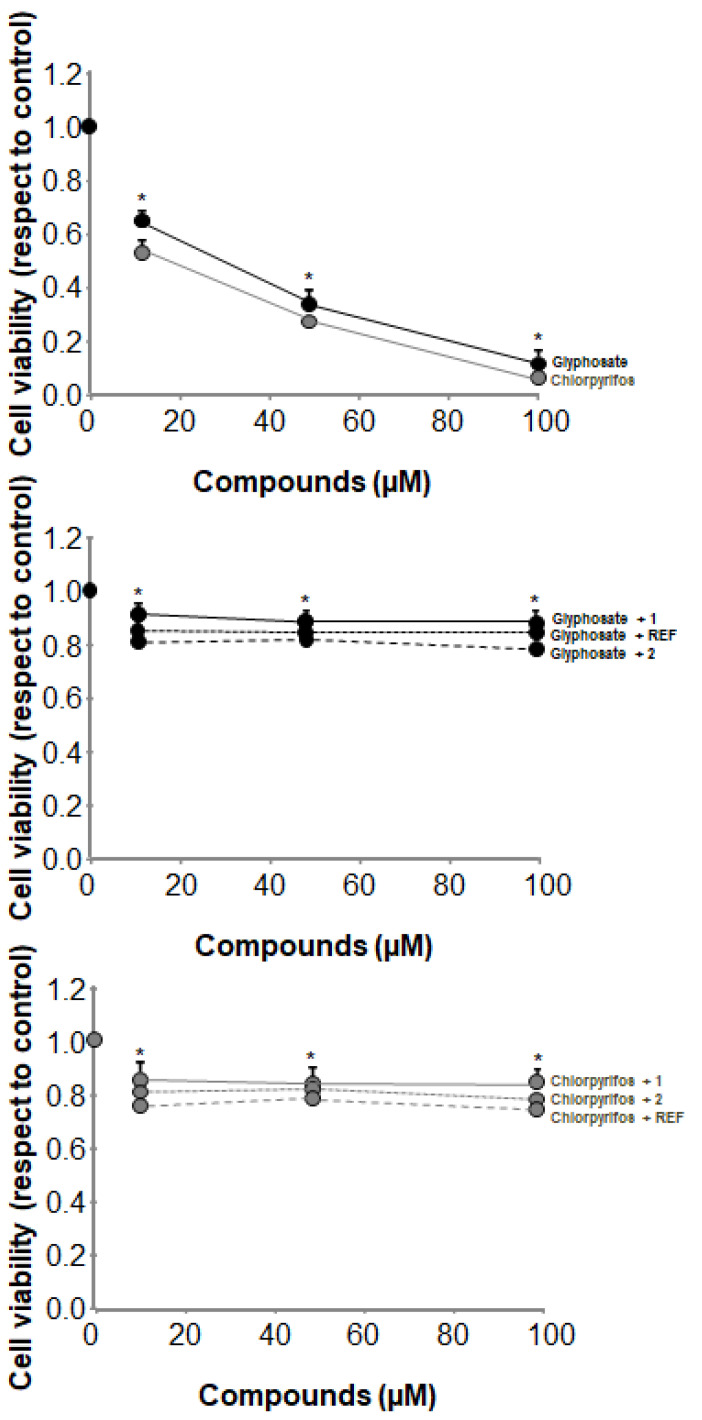
Effects of glyphosate and chlorpyrifos as individual compounds and in combination with compounds **1**, **2**, and resveratrol (REF) (all at the concentration level of 50 µM) on BEAS-2B cell viability (* Significance non-treated vs. chlorpyrifos or glyphosate + compounds under investigation).

As a confirmation of the real protective properties on mitochondrial functions by the compounds herein under investigation, with particular reference to auraptene **1** and 7-isopentenyloxycoumarin **2**, in turn resulting in an effective protective effect on BEAS-2B line viability, we measured this parameter in these cells exposed to either glyphosate or chlorpyrifos alone or in combination with samples **1, 2**, having resveratrol again as the reference using the MTT test. Results are reported in Figure 6.

As expected, both glyphosate and chlorpyrifos induce a massive decrease in cell viability, in line with have been reported several times in the literature. The administration to cells of auraptene **1**, 7-isopentenyloxycoumarin **2**, and resveratrol (all at the concentration of 50 μM) provided an appreciable protective effect, as observed for mitochondrial functions, for which a significant increase in cell viability has been recorded. This last set of experiments confirmed that the positive modulatory effects by compounds **1** and **2** on mitochondria function and activity represents an overall benefit for the whole cell.

## 4. Discussion

In this manuscript we basically studied the extent of protection on mitochondria functions stressed by the application of the organophosphate pesticides. glyphosate and chlorpyrifos, by a panel of five oxyprenylated phenylpropanoids comprising two coumarins (auraptene **1** and 7-isopentenyloxycoumarin **2**) and cinnamic (4′-isopentenyloxycinnamoc acid **3**) and ferulic acids (boropinic acid **4** and 4′geranyloxyferulic acid **5**) derivatives. The compounds selected are the most representative and most abundant *O-*prenylphytochemicals occurring in nature and are compounds that have also received the most detailed investigations. Previous studies about the interaction of oxyprenylated phenylpropanoids and mitochondria reported the activity of only auraptene **1**. Data in the literature concerning these appears to be somewhat controversial. Thus, the first observation to this concern dates back to 2006, when Jeong and coworkers reported that compound **1**, extracted from the aerial parts of *Dictamnus albus* L. (Fam. Rutaceae, common names “burning bush”, “dittany”, or “fraxinella”) showed a slight and potently selective inhibitory effect against monoamino oxidase (MAO)-B (IC_50_ = 0.6 μM,) compared to MAO-A (IC_50_ = 34.6 μM), both representing two flavin-containing enzymes located in the outer mitochondrial membrane [49]. However, these authors did not refer further about potential applications both in in vitro and in vivo pharmacological models. In 2007 Jun and coworkers highlighted that this *O-*prenylcoumarin, extracted from the leaves of the Chinese medicinal plant *Zanthoxylum schinifolium* Siebold & Zucc. (Fam. Rutaceae, common name “mastic-leaf prickly ash”), was able to induce apoptosis in human acute leukemia Jurkat T cells. However, this effect was given by an interaction of auraptene with endoplasmic reticulum. This in turn stress-mediated the activation of resident caspases 12 and 8 and led to the subsequent and final involvement of mitochondrial structures by the release of cytochrome c and activation of caspases 9 and 3. This cascade of events provided Jurkat T cells apoptosis. Consequently, the paper by Jun and coworkers described only an indirect interaction between auraptene **1** and mitochondria [50]. In 2013 Nagle and coworkers indicated that the biomolecular mechanism underlying this effect consisted in the inhibition of hypoxia-induced HIF-1α activation, thus suppressing mitochondria-mediated hypoxic signaling [51]. A similar finding was reported in 2015 by Jang and coworkers [52]. These authors reported that compound **1** acted as a mitochondrial poison in RCC4 (human renal carcinoma) cancer cell lines and found that auraptene abolished RCC4 cells motility through a marked decrease of mitochondrial respiration and of the expression of glycolytic pathway-related genes expression. Furthermore compound **1** strongly disrupted vascular endothelium growth factor (VEGF)-induced angiogenesis both in vitro and in vivo. Aurpatene also impeded the hypoxia-inducible factor 1a (HIF-1a), well known nowadays to play a key role in the metabolism of cancer cells, as well as migration, and, more importantly, angiogenesis. This process in particular is stably expressed and active in RCC4 cells due to a resident genetic mutation in the von Hippel–Lindau (VHL) tumor-suppressor protein. In particular, such a blockade could contribute to the observed suspension of RCC progression. Thus, results and outcomes form this last study showed that auraptene may represent a beneficial compound being able to counteract cancer progression, while in the one reported by Nagle and coworkers, the same effects by compound **1**, were seen in a negative light with regard to its claimed cancer chemo-preventive properties. In 2019, Jang and coworkers described for the first time results in which auraptene exerted protective effects against the inhibition of mitochondrial respiration [53]. In particular, these authors pointed out that compound **1** markedly protected dopaminergic neurons of substantia nigra from rotenone (another well-known pesticide and mitochondrial poison) evoked a boost of ROS and overall mitochondrial oxidative damage. Auraptene also massively induced the expression of antioxidant enzymes Jang and coworkers tested auraptene also in vivo. In detail, **1** was assessed recording the expression of tyrosine hydroxylase (TH), the rate-limiting step enzyme in the biosynthesis of dopamine, in the striatum and substantia nigra of 1-methyl-4-phenyl-1,2,3,6-tetrahydropyridine (MPTP)-induced Parkinson’s disease model mice and behavioral changes after injection of the same auraptene. Treatment with compound **1** largely ameliorated movement, in manner consistent with the observed increase in the number of dopaminergic neurons in the substantia nigra. Data by Jang and coworkers led to the hypothesis that auraptene may target dual pathogenic mechanisms by enhancing mitochondrial respiration and attenuating ROS production. These findings could account for the observed ameliorating effects of oxyprenylated coumarin in subjects affected by Parkinson’s disease. Similar beneficial effects by compound **1** were recorded in 2021 by Akashi and coworkers [54]. These authors found that an extract of the Japanese fruit *Citrus* x *hassaku* Hort. Tanaka enriched in auraptene (>80%) strongly increased the expression of the proliferator-activated receptor γ coactivator-1α (PGC-1α) in skeletal muscle. In particular, auraptene, administered in the diet for five weeks with the enriched extracts to C57BL/6J mice, provided a large increase of PGC-1α and overall mitochondrial biogenesis and muscle fibers to oxidative fibers. Furthermore, the auraptene-enriched extract increased the expression of the protein sirtuin 3, of phosphorylated AMP-activated protein kinase (AMPK), and of the transcriptional activity of PGC-1α. Considered as a whole, the findings by Akashi and coworkers led tem to hypothesize that auraptene, as part of the phytocomplex from *C. hassaku*, may mediate PGC-1α expression in skeletal muscles and may serve as a dietary supplement to prevent metabolic disorders, as previously demonstrated in other in vitro and in vivo models. Based on the observed results, which can be summarized in an increased metabolism of sugars and lipids and their catabolism at the mitochondrial level, the authors stated that auraptene could represent an efficient means for the dietary chemoprevention of metabolic disorders. Finally, the last acquisition in the literature was reported in the same year by Lee and coworkers, who described how auraptene was able to enhance junction assembly in cerebrovascular endothelial cells by exerting beneficial effects against mitochondrial stress through the activation of antioxidant enzymes, in the same way as observed by Jang and coworkers, and the mitochondrial unfolded protein response (mtUPR) [55]. In particular, these authors highlighted that the increase of the mRNA expression of antioxidant enzymes induced by auraptene provided a parallel increase of the expression of the junctional proteins occluding, zonula occludens-1 (ZO-1), and vascular endothelial (VE)-cadherin. Compound **1** was also able to depolarize mitochondrial membrane potential leading to the activation of mtUPR. The capacity by auraptene to protect the brain against ischemia was also assessed using cells deprived of oxygen and glucose. Thus, pretreatment of these cells with compound 1 prevented the damage to junctional proteins, including occludin, claudin-5, ZO-1, and VE-cadherin. A stress resilience response regulated by increased levels of mRNAs related to Activating Transcription Factor (ATF) 5, Lon Protease (LONP) 1 and Heat Shock Protein (HSP) 60 was also observed. As a conclusion, Lee and coworkers stated that auraptene is an efficient promoter of resilience against oxidative stress at a mitochondrial level, helping to maintain intact barriers in cerebrovascular endothelial cells.

This brief survey of the already reported literature acquisitions seems to indicate how apparently contrasting results about the interaction of an oxyprenylated phenylproanoid such as auraptene with mitochondria have been reported in the literature. In some cases, disruptive outcomes have been recorded, while in the most recent studies, a protective role has been highlighted. However, this discrepancy may derive from the fact that different targets at a mitochondrial level have been selected. When assayed on HIF-1a and associated cell responses, auraptene led to negative effects, leading to huge disorders in mitochondrial functions. In this case, no stress stimuli have been administered to mitochondria. For the investigations reported in the literature between 2019 and 2021, such a stimulus (represented by a pesticide or ROS) was applied. In this context, compound **1** acted to improve the content of antioxidant enzymes, to increase the extent of biogenesis of mitochondria, and finally to restore the mitochondrial potential as the main biomolecular pathways. In line with what has been reported earlier and just mentioned, results obtained in the present study are a confirmation of the protective effects of auraptene on mitochondria when exposed to stress stimuli, represented herein by the organophosphates pesticides chlorpyrifos and glyphosate. Such positive effects have been exerted not only by auraptene itself, but also by other oxyprenylated coumarins such as 7-isopentenyloxycoumarin. Such findings have been reported in the literature herein for the first time. On the other hand, p-coumaric and ferulic acids derivatives **3–**5 cannot be claimed as protective agents on mitochondrial functions, as the recorded results are rather controversial. Thus, in terms of structure-activity relationship considerations, the presence of a coumarin core may be a determinant for the observed effects. Results reported in the present investigation may represent an effective boost to investigating the effects of a wider panel of naturally occurring oxyprenylated coumarins as protective agents for mitochondria stressed by mitogen stimuli.

Both auraptene **1** and 7-isopentenyloxycoumarin have been demonstrated to exert neuroprotective effects, also in terms of amelioration of symptoms and clinical significance associated to the development and progress of Parkinson’s disease [13,14]. In the meantime, pesticides, including glyphosate [56] and chlorpyrifos [57], have been claimed to be possible causes of neurodegenerative diseases in humans upon chronic exposure with the effective involvement of mitochondria and associated disruption. Data reported in the present paper are a valid contribution in trying to explain and clarify the mechanism of action underlying the observed and reported properties of compounds 1 and **2** as neuroprotective and anti-Parkinson’s disease agents.

## 5. Conclusions

As a final consideration, the potency displayed by compounds **1** and **2** may shed more light on the molecular basis of the beneficial effects of auraptene and structurally related oxyprenylated phenylpropanoids. Thus, mitochondria can be effectively included in the set of biological targets triggered by this group of natural products. Since the phytochemicals investigated in this study are contained in several medicinal, healthy, and food plants, that in turn are widespread and consumed in many regions of the world, the findings described in this manuscript can help in identifying new beneficial remedies for human health in terms of prevention and therapy, as well as pointing the way to a new category of nutraceuticals. Studies to deepen the knowledge about the herein investigated oxyprenylated phenylpropanoids and other naturally occurring compounds belonging to the same group are now ongoing in our laboratories.

## Data Availability

Data is contained within the manuscript.
